# *Mentha spicata* Essential Oil: Chemical Composition, Antioxidant and Antibacterial Activities against Planktonic and Biofilm Cultures of *Vibrio* spp. Strains

**DOI:** 10.3390/molecules200814402

**Published:** 2015-08-07

**Authors:** Mejdi Snoussi, Emira Noumi, Najla Trabelsi, Guido Flamini, Adele Papetti, Vincenzo De Feo

**Affiliations:** 1 Laboratoire de Traitement des Eaux Usées, Centre de Recherches et des Technologies des Eaux (CERTE), Technopole de Borj-Cédria, BP 273, 8020 Soliman, Tunisia; E-Mail: snmejdi@yahoo.fr; 2 Laboratory of Transmissible Diseases and Biological Active Substances, University of Monastir, Avenue Avicenne, 5000 Monastir, Tunisia; E-Mail: emira_noumi@yahoo.fr; 3 Laboratoire de Biotechnologie de l’Olivier, Centre de Biotechnologie de Borj Cédria, BP 901, 2050 Hammam Lif, Tunisia; E-Mail: trabnajla@yahoo.fr; 4 Department of Pharmacy, University of Pisa, Via Bonanno, 6, 56126 Pisa, Italy; E-Mail: guido.flamini@farm.unipi.it; 5 Department of Drug Sciences, University of Pavia, Viale Taramelli, 12, 27100 Pavia, Italy; E-Mail: adele.papetti@unipv.it; 6 Department of Pharmacy, University of Salerno, Via Giovanni Paolo II, 132, Fisciano 84084, Salerno, Italy

**Keywords:** *Mentha spicata*, chemical composition, antioxidant activity, anti-*Vibrio* activity

## Abstract

Chemical composition, antioxidant and anti-*Vibrio* spp. activities of the essential oil isolated from the aerial parts of *Mentha spicata* L. (spearmint) are investigated in the present study. The effect of the essential oil on *Vibrio* spp. biofilm inhibition and eradication was tested using the XTT assay. A total of 63 chemical constituents were identified in spearmint oil using GC/MS, constituting 99.9% of the total identified compounds. The main components were carvone (40.8% ± 1.23%) and limonene (20.8% ± 1.12%). The antimicrobial activity against 30 *Vibrio* spp. strains (16 species) was evaluated by disc diffusion and microdilution assays. All microorganisms were strongly affected, indicating an appreciable antimicrobial potential of the oil. Moreover, the investigated oil exhibited high antioxidant potency, as assessed by four different tests in comparison with BHT. The ability of the oil, belonging to the carvone chemotype, to inhibit or reduce *Vibrio* spp. biofilm warrants further investigation to explore the use of natural products in antibiofilm adhesion and reinforce the possibility of its use in the pharmaceutical or food industry as a natural antibiotic and seafood preservative against *Vibrio* contamination.

## 1. Introduction

Tunisia has a remarkable floral and cultural diversity, with a number of endemic plants due to the variable climate and the high number of ecological zones [1]. A great number of plant species are used for traditional and medicinal purposes. Aromatic/medicinal plants and spices have been used for thousands of years as medicines due their bioactive compounds [2]; moreover, many aromatic plants growing wild or cultivated in Tunisia are still to be investigated for their chemical composition and biological activities and their potential use in perfumery, food preparation and conservation, and pharmaceutical preparations.

The Lamiaceae family consists of more than 4000 species in 200 genera. Many genera within this family are medicinal plants useful in human disease therapy as well as in food, both raw and cooked. Many lamiaceous species contain essential oils that show biological activity against many bacterial and fungal pathogens.

The genus *Mentha* includes 25–30 species that grow under cultivation from tropical to temperate climate of America, Europe, China, Brazil, India, Australia, and South Africa. *M. arvensis* L. (corn mint), *M*. *x piperita* L. (peppermint), *M*. *citrata* Ehrh. (bergamot mint), *M*. *longifolia* L. (wild mint), and *M*. *spicata* L. (spearmint) are the main species cultivated in temperate, Mediterranean, and subtropical regions [3,4,5,6].

In Tunisia, the *Mentha* genus is represented by five species: *M*. *pulegium* L., *M*. *longifolia*, *M*. *spicata*, *M*. *aquatica* L., and *M*. *rotundifolia* (L.) Huds., which are well represented in the north of the country and in humid zones [1]. These species show considerable chemical diversity in their essential oil composition and are considered industrial crops as they produce a number of commercially valuable volatile oils containing complex mixtures of monoterpenoids and are extensively used in the pharmaceutical, food, flavor, cosmetics, beverages, and allied industries [7]. India fulfills 80% of the total mint global demand with a production of 16,000 tons of mint oil [8]. A number of medicinal uses in the Mediterranean countries for the “mint” taxa were documented by Boukef and co-workers [9], who scored 39 uses for both *M*. *spicata* and *M*. *pulegium*, with 16 uses in Cyprus and only three uses in Tunisia (abortifacient, or treatment of stomach ailments and toothache).

*M*. *spicata* L. (spearmint) is an herbaceous rhizomatous perennial plant growing 30–100 cm tall, with variably hairless to hairy stems and foliage, and a wide-spreading fleshy underground rhizome. The leaves are 5–9 cm long and 1.5–3 cm broad, with a serrated margin. The stem is square-shaped, a trademark of the mint family of herbs. Spearmint produces flowers in slender spikes, each flower pink or white and 2.5–3 mm long and broad. The leaves are popularly used as a tea flavoring agent and the whole plant is used as a carminative. The fresh and dried plants and their essential oils are widely used in the food, cosmetic, confectionary, chewing gum, toothpaste, and pharmaceutical industries [10]. This species is also often used in Indian and Italian cuisine and usually added fresh or dried to fish and shellfish plates before or after cooking. *M. spicata* possesses several biological activities and is used in folkloric medicine as a carminative, antispasmodic, diuretic, antibacterial, antifungal, and antioxidant agent, and for treatment of colds and flu, respiratory tract problems, gastralgia, hemorrhoids, and stomachache [2,11,12,13].

Few studies have investigated the effects of spices and herbs against marine pathogenic *Vibrio* spp. strains [6,14,15,16]. Many spices and herbs have been used for taste and preservation of various foods in world cuisine and could be introduced to raw and lightly cooked seafood. Spices and herbs are generally used for food subject to bacterial contamination [17]. The Japanese custom of eating raw and lightly cooked seafood is increasingly popular in Europe, the United States, and other Asian countries, and seems to have also been adopted in many countries around the world with the globalization of food. *Wasabia japonica* Matsum. (Brassicaceae) is traditionally used when eating raw fish such as sushi to confer protection against several bacterial strains including *V. parahaemolyticus* and is believed to contribute to the safety of eating raw seafood [18].

The aims of this work were (1) to study the chemical composition of Tunisian *M. spicata* harvested from Soliman Tunisian locality (Nabeul), which is commonly used in Tunisian kitchens to dress fish and shellfish dishes; (2) to evaluate its possible antioxidant and antimicrobial effects against several pathogenic *Vibrio* spp. isolated from seawater and fish and associated with human infection due to consumption of raw or undercooked sea products; and (3) to evaluate its ability to prevent and eliminate *Vibrio* spp.

## 2. Results and Discussion

### 2.1. Essential Oil Composition

The composition of the essential oil of *M. spicata* is presented in Table 1. Thirty-four compounds were identified, representing 99.9% of the total compounds. The oil contains 50.6% oxygenated monoterpenes, 45.1% monoterpene hydrocarbons, and 2.8% of sesquiterpene hydrocarbons. The main constituents were carvone (40.8% ± 1.23%) and limonene (20.8% ± 1.12%), followed by 1,8-cineole (17.0% ± 0.60%), β-pinene (2.2% ± 0.25%), *cis*-dihydrocarvone (1.9% ± 0.49%), and dihydrocarveol (1.7% ± 0.31%). The oil yield of this Tunisian variety of spearmint was 1.1% and it can be ascribed to the carvone/limonene chemotype. In fact, there is large variation in the chemical composition of *M. spicata*, wild as well as cultivated, around the world. Indeed, a series of chemotypes have been described in previous studies, with prevalence of pulegone, carvone, linalool, piperitone, piperitone oxide, menthone/isomenthone, pulegone/menthone/isomenthone, and pulegone/piperitone [19,20,21]. Moreover, carvone-rich essential oils are widely used as spices in the flavor and fragrance industries in Europe [20]. Other *Mentha* species, like *M. longifolia*, *M. suaveolens* Ehrh. [22], *M. viridis* L. [23], and *M. haplocalyx* Briq. [24], are characterized by the prevalence of carvone and limonene.

**Table 1:** Chemical composition of Tunisian *M. spicata* essential oil.

Components	I.r.i. ^a^	Percent
α-pinene	941	1.4 ± 0.17
camphene	955	0.2 ± 0.06
sabinene	977	1.4 ± 0.06
β-pinene	982	2.2 ± 0.25
myrcene	993	1.1 ± 0.15
3-octanol	994	1.0 ± 0.21
*p*-cymene	1028	0.8 ± 0.06
limonene	1032	20.8 ± 1.12
1,8-cineole	1034	17.0 ± 0.60
*(Z)*-β-ocimene	1042	0.2 ± 0.06
*cis*-sabinene hydrate	1070	1.6 ± 0.15
linalool	1101	0.4 ± 0.12
*cis-p*-menth-2-en-1-ol	1123	0.1 ± 0.00
*cis*-limonene oxide	1138	0.1 ± 0.06
*trans*-limonene oxide	1141	0.1 ± 0.00
borneol	1171	0.1 ± 0.06
δ-terpineol	1172	0.4 ± 0.12
4-terpineol	1179	1.3 ± 0.26
α-terpineol	1191	0.5 ± 0.10
dihydrocarveol	1194	1.7 ± 0.31
*cis*-dihydrocarvone	1195	1.9 ± 0.49
*trans*-carveol	1219	0.4 ± 0.06
*cis*-carveol	1231	0.6 ± 0.15
pulegone	1239	0.3 ± 0.06
carvone	1244	40.8 ± 1.23
isobornyl acetate	1287	0.1 ± 0.00
*iso*-dihydrocarveol acetate	1327	0.2 ± 0.06
β-bourbonene	1385	0.9 ± 0.17
β-elemene	1392	0.3 ± 0.06
β-caryophyllene	1419	1.2 ± 0.25
germacrene D	1481	0.2 ± 0.06
germacrene A	1506	0.2 ± 0.15
spathulenol	1578	0.1 ± 0.00
caryophyllene oxide	1582	0.3 ± 0.06
monoterpene hydrocarbons		45.1
oxygenated monoterpenes		50.6
sesquiterpene hydrocarbons		2.8
oxygenated sesquiterpenes		0.4
others		1.0
total identified		99.9

^a^ l.r.i.: Linear Retention Index.

The differences in oil content and composition may be attributed to factors related to ecotype, phenophases, temperature, relative humidity, photoperiod, irradiance, genotype, and agronomic conditions (harvesting time, plantage, crop density). In the north Indian plains carvone content varies between 45.9% and 77.1% [25]. The percentage of carvone also varies in the essential oil of spearmint growing in different countries, e.g., Egypt (46.4%–68.55%) [26,27], Canada (59%–74%) [28], Colombia (61.53%) [29]; Turkey (78.35%–82.2%) [20,30], China (55.45%–74.6% [31], Bangladesh (73.2%) [32], Algeria (59.4%) [33], and Morocco (29%) [34]. Lower amounts of carvone were reported in the spearmint essential oil from Iran (22.4%) [35]. A linalool-rich chemotype (82.8%) was also reported from Turkey [19]. In another report on *M. spicata* essential oil from Iran, α-terpinene (19.7%), piperitone oxide (19.3%), isomenthone (10.3%), and β-caryophyllene (7.6%) were reported as major components [36], while the Serbian *M. spicata* essential oil is characterized by menthone (21.9%), carvone (49.5%), limonene (5.7%), 1,8-cineole (3%), and β-mycrene (2.3%) as its main components [37]. Chauhan *et al.* [38] reported that the fresh plants from the northwest Himalayan region (China) have 0.57% essential oil on a fresh weight basis, with a total of 20 compounds constituting 96.24% of the total essential oil. The main components were carvone (76.65%), limonene (9.57%), cis-dihydrocarvone (2.04%), and 1,8-cineole (1.93%). In Egypt, Omar *et al.* [39] reported that menthone (32.43%), 1,8-cineole (18.79%), *cis*-*iso* pulegone (16.65%), pulegone (10.01%), β-pinene (7.12%), α-cadinol (5.30%), and α-pinene (5.03%) were the main components of the *M. spicata* essential oil harvested from the Zagazig Region.

In Tunisia, the only study on the chemical composition of *M. spicata* essential oil harvested from Sfax (the south of the country) reported that the essential oil was characterized by 92.18% oxygenated monoterpenes, 2.74% monoterpene hydrocarbons, and 3.1% sesquiterpenes [40], and the main components were identified as l-menthone (32.74%), pulegone (26.67%), 1,8-cineole (11.16%), and menthol (11.42%). In *M. spicata* essential oil harvested from four Turkish provinces, with different climatic factors and soil mixture, limonene (3.2%–5.21%) and β-phellandrene (1.31%–2.55%) were the major monoterpene hydrocarbons; *trans*-caryophyllene (5.23%–8.01%) and germacrene D (3.08%–5.32%) were the major sesquiterpenes [37].

Recently, Zhao *et al.* [41] studied the variation in the chemical composition of eight accessions of *M. spicata* essential oil originating from seven provinces in China. The essential oil yield varied from 0.5% to 0.8% of the dry weight and these accessions were grouped into the single chemotype carvone (46.7%–65.41%). The percentage of oxygenated monoterpenes ranges between 87.1% and 94%. In the same year, Padalia *et al.* [7] studied the chemical composition of 16 cultivars of *Mentha* from the western Himalayan region (China) and reported that carvone (51.3%–65.1%), limonene (15.1%–25.2%), β-pinene (1.3%–3.2%), and 1,8-cineole (≤0.1%–3.6%) are the major components in five cultivars of *M. spicata*.

### 2.2. Antioxidant Activity

Four antioxidant assays have been used to evaluate the possible antioxidant properties of the spearmint essential oil, including DPPH radical scavenging activity, superoxide anion scavenging, reducing power, and antioxidant assay using β-carotene linoleate system. The results obtained (Table 2) confirm previous reports about the importance of essential oils as natural antioxidants and their possible role in the protection of human health. Organic extracts of *Mentha* species have been found to have antioxidant and antiperoxidant properties due to the presence of eugenol, caffeic acid, rosmarinic acid, and α-tocopherol [42]. An aqueous extract of *M. x piperita* provides protection against radiation-induced chromosomal damage in the bone marrow of mice by decreasing serum acid phosphatase and increasing serum alkaline phosphatase [43].

**Table 2:** Antioxidant activity of *M. spicata* essential oil.

Activity (µg/mL)	Spearmint Oil	BHT	BHA	Ascorbic Acid	EDTA
DPPH IC_50_	3.08 ± 0.07	11.48 ± 0.02	-	-	-
Reducing Power EC_50_	2.49 ± 0.07	-	-	37.53 ± 0.39	-
Chelating Power IC_50_	6.33 ± 0.12	-	-	-	32 ± 0
β-Carotenes IC_50_	6.4 ± 0.07	-	48.00 ± 0.50	-	-

The antioxidant ability of the spearmint oil was measured by the bleaching of a purple methanol solution of DPPH. This spectrophotometric assay uses the stable radical 2,2′-diphenyl-1-picrylhydrazyl (DPPH) as a reagent. The IC_50_ value for spearmint essential oil was 3 µg/mL in comparison to 11.5 µg/mL for the standard compound, BHT. Dhifi *et al.* [40] reported that the IC_50_ value of the *M. spicata* essential oil harvested from south of Tunisia (chemotype menthone/pulegone) was about 10 µg/mL. Some compounds such as as phenylpropanoids, monoterpenes, and oxygenated sesquiterpenes are reported to have oxidation inhibition capacity [44]. This interesting biological activity can be explained by the presence in our oil of the monoterpenes limonene, terpinolene, γ-terpinene, 1,8-cineole, and carvone.

Dorman *et al.* [45] reported that different *Mentha* extracts were capable of scavenging DPPH radicals in the following decreasing order: *M. piperita, M. dalmatica*, and *M. spicata.* Mata *et al.* [46] stated that an ethanolic extract of *M. spicata* showed lower antioxidant activity than that of BHT. Arumugam *et al.* [42] indicates that the ethyl acetate fraction of an ethanolic extract of *M. spicata* showed higher antioxidant activity against ABTS•+ than the hexane and chloroform fractions. This activity is due to the high content of phenolic compounds, which could be most effective in protecting the body against various oxidative stressors.

Nickavar and colleagues [47] evaluated the antioxidant and free radical scavenging properties as well as the phenolic content of the ethanol extract from five *Mentha* species (*M. longifolia* (L.) Huds., *M. piperita* L., *M. pulegium* L., *M. rotundifolia* (L.) Huds., and *M. spicata* L.) using two different methods, 2,2′-diphenyl-1-picrylhydrazyl radical (DPPH•) and 2,2′-azinobis (3-ethylbenzothiazoline-6-sulfonic acid) radical (ABTS•+). *M. piperita* exhibited the strongest activity as a DPPH• scavenger. On the other hand, all extracts were active in the ABTS•+ assay. The highest scavenging activity was observed for *M. piperita* [IC_50_ = 13.32 (12.12–14.64) μg/mL], and the lowest for *M. spicata* [IC_50_ = 87.89 (81.66–94.59) μg/mL]. The IC_50_ (DPPH•) values of the extracts increased in the following order: *M. piperita* < *M. pulegium* < *M. rotundifolia* ≤ *M. longifolia* < *M. spicata*. *M. piperita* showed the highest total phenolic content (433.60 ± 19.62 μg/mg), whereas *M. spicata* had the lowest (150.9 ± 5.14 μg/mg). The total phenolic content of the extracts in decreasing order was: *M. piperita* > *M. pulegium* ≥ *M. rotundifolia* ≥ *M. longifolia* > *M. spicata*. According to the results obtained by Kizil *et al.* [12], the highest radical scavenging activity was observed in the following order; ascorbic acid > *M. piperita* > BHA > *M. spicata.* The free radical scavenging activity of the two mint species showed that the essential oils of *M. spicata* (IC_50_ = 77.40 µg/mL) are more effective than those of *M. piperita* (IC_50_ = 60.41 µg/mL).

Recently, Naidu *et al.* [48] reported that the total phenolic component of a crude methanolic extract of *Mentha spicata* was found to be 27.26 ± 0.62 mg/g gallic acid equivalent. The DPPH radical scavenging activity was found to increase with increasing concentrations and was found to be 54.84% ± 0.57% with an IC_50_ value of 25.2 µg/mL. In the same year, Martins *et al.* [49] studied the chemical composition and antioxidant activities of spearmint essential oil harvested from Portugal and reported the identification of 30 components that constitute 87.7% of the total composition. Oxygenated monoterpenes (46.3%), monoterpene hydrocarbons (25.5%), and sesquiterpene hydrocarbons (14.1%) were found to be the major constituent groups, with the monoterpere carvone (41.1%) as the main constituent. This essential oil showed antioxidant activity both by DPPH radical scavenging method (31.45%) and by system β-carotene/acid linoleic method (14.89%).

### 2.3. Anti-Vibrio *spp.* Activity

The antibacterial activity of *M. spicata* L. essential oil tested against 30 *Vibrio* spp. microorganisms was examined both qualitatively (inhibition zone diameter) and quantitatively (MIC and MBC values). Moreover, its potency to inhibit and eradicate the biofilm formed on polystyrene surface (XTT assay) was tested in the present study (Table 3). The results showed that the studied essential oil had substantial anti-*Vibrio* spp. activity with zones of growth inhibition (mm) scored in Mueller-Hinton agar-1% NaCl ranging from 7 ± 0 mm for *V. alginolyticus* ATCC 17749 to 21.33 ± 0.58 mm for *V. alginolyticus* (Malaga, Spain). Interestingly, *V. alginolyticus* and *V. vulnificus* strains isolated from *Dicentrarchus labrax*, *Sparus aurata*, and *Mytilus edulis* were more sensitive to the spearmint essential oil than the two *V. parahaemolyticus* strains associated with *M. edulis* from the Bizerte Lagoon (10.00 and 11.67 mm, respectively). The essential oil bacteriostatic activity was confirmed by the low MIC values, ranging from 0.023 to 0.047 mg/mL, while higher concentrations were needed for a bactericidal action (MBC values ranging from 0.75 to 12 mg/mL).

**Table 3:** Growth inhibition zone (IZ, mm), MIC, MBC for *M. spicata* essential oil.

Microorganisms	*M. spicata* Essential Oil		
	GIZ ± SD	MIC	MBC
*V. cholerae* ATCC 9459	13.67 ± 0.58 ^f,g^	0.023	12
*V. cholerae* (Granchi, Ancona)	14.67 ± 0.58 ^f^	0.023	12
*V. vulnificus* ATCC 27562	13.33 ± 0.58 ^g^	0.023	6
*V. vulnificus S_5_* (*D. labrax,* Chebba)	14.67 ± 0.58 ^e,f^	0.047	6
*V. vulnificus V_30_* (*S. aurata,* Hergla)	13.67 ± 0.58 ^f,g^	0.023	6
*V. parahaemolyticus* ATCC 17802	14.67 ± 0.58 ^e,f^	0.023	12
*V. parahaemolyticus* ATCC 43996	15.67 ± 0.58 ^d^	0.023	12
*V. parahaemolyticus* I_12_ (Seawater, Italy)	17.33 ± 0.58 ^c^	0.023	12
*V. parahaemolyticus* I_22_ (Seawater, Italy)	19.33 ± 0.58 ^b^	0.047	6
*V. parahaemolyticus* (Malaga, Spain)	14.33 ± 0.58 ^f,g^	0.047	3
*V. parahaemolyticus S*_949_ (*M. edulis*, Bizerte)	10 ± 0 ^i^	0.047	12
*V. parahaemolyticus S*_950_ (*M. edulis*, Bizerte)	11.67 ± 0.58 ^h^	0.023	12
*V. alginolyticus* ATCC 33787	18.67 ± 0.58 ^b^	0.023	24
*V. alginolyticus* ATCC 17749	7 ± 0 ^j^	0.047	12
*V. alginolyticus* (Malaga, Spain)	21.33 ± 0.58 ^a^	0.047	12
*V. alginolyticus S_6_* (*D. labrax,* Chebba)	12.33 ± 0.58 ^h^	0.023	1.5
*V. alginolyticus S_7_* (*M. edulis,* Bizerte)	13.67 ± 0.58 ^f,g^	0.023	0.75
*V. alginolyticus S_8_* (*S. aurata,* Hergla)	14.33 ± 0.58 ^f,g^	0.023	6
*V. furnisii* ATCC 35016	14.33 ± 0.58 ^f,g^	0.047	6
*V. cincinnatiensis* ATCC 35912	13.67 ± 0.58 ^f,g^	0.023	12
*V. proteolyticus* ATCC 15338	14.33 ± 0.58 ^f,g^	0.023	12
*V. natrigens* ATCC 14048	13.67 ± 0.58 ^f,g^	0.023	12
*V. mimicus* ATCC 33653	15.67 ± 0.58 ^d^	0.047	12
*V. fluvialis* ATCC 33809	13.33 ± 0.58 ^g^	0.023	12
*V. anguillarum* (Malaga, Spain)	13.67 ± 0.58 ^f,g^	0.023	12
*V. carchariae* ATCC 35084	9.67 ± 0.58 ^i^	0.023	12
*V. harveyii* ATCC 18293	13.67 ± 0.58 ^f,g^	0.047	12
*V. diazotrophicus* ATCC 33466	14.67 ± 0.58 ^e,f^	0.047	12
*V. tapetis* CECT 4600^T^	14.57 ± 0.58 ^e,f^	0.023	12
*V. splendidus* ATCC 33125	17.67 ± 0.58 ^c^	0.047	12
*A. hydrophila* ATCC 7966^T^	14.67 ± 0.58 ^e,f^	0.023	12

(a, b, c, d, e, f, g, h, i, j): Means followed by the same letters are not significantly different at *P* = 0.05 based on Duncan’s multiple range tests; GIZ ± SD: Inhibition zone around the discs impregnated with of essential oil (10 mg/disk), expressed as mean of three replicates (mm); SD: standard deviation. MIC: Minimal Inhibitory Concentration; MBC: Minimal Bactericidal Concentration expressed as (mg/mL).

In a previous studies [15,16], we tested the effect of five essential oils from plants frequently used in food preparation in Tunisia, including two *Mentha* species (*M. pulegium* and *M. longifolia*), *Thymus vulgaris* L., *Rosmarinus officinalis* L. (Lamiaceae), and *Sygyzium aromaticum* (L.) Merr. & L. M. Perry (Myrtaceae), against several *Vibrio* spp. strains (including those tested in the present study, Table 4). We found that the thyme oil possess high levels of anti-*V. parahaemolyticus* strains with a diameter of inhibition zone ranging from 14 to 28 mm [15] and low MIC and MBC values (MIC 0.078–0.156; MBC > 0.31–1.25 mg/mL). Compared to the two mint species previously tested, *M. spicata* essential oil was more active on *V*. *alginolyticus* (ATCC 33787 and ATCC 17749), *V*. *parahaemolyticus* (ATCC 17802 and ATCC 43996), *V*. *vulnificus* ATCC 27562, and *V*. *fluvialis* ATCC 33809, with a diameter of growth inhibition zone ranging from 13.33 to 18.67 mm (Table 4). Hajlaoui *et al.* [16] studied the effects of cumin oil on *Vibrio* spp. strains and found that the diameters of growth inhibition zones ranged from 11 mm (*V*. *alginolyticus* ATCC 33787) to 23 mm (*V. cholerae* ATCC 9454). The same authors reported that the MIC and MBC values indicated that the essential oil of cumin was efficient against *Vibrio* spp. strains (with MIC values ranging between 0.078 and 0.31 mg/mL) and low concentrations of cumin essential oil were sufficient to inhibit the growth of several pathogenic *Vibrio* species including, *V. cholerae*, *V. parahaemolyticus*, and *V. vulnificus*.

**Table 4:** Comparison between the growth inhibition zone (GIZ, mm), MIC (mg/mL) and MBC (mg/mL) obtained with *Eugenia caryophyllata*, *Thymus vulgaris*, *Rosmarinus officianalis*, *Cuminum cyminum, M. pulegium, M. longifolia*, and *M. spicata* essential oils against some *Vibrio* spp. strains.

*Vibrio* strain		1	2	3	4	5	6
E. caryophyllata *	GIZ	11.33 ± 0.57	10.66 ± 0.57	13.66 ± 0.57	12.33 ± 0.57	11.66 ± 0.57	10 ± 0
	MIC	0.15	0.31	0.156	0.156	0.156	0.156
	MBC	1.25	2.5	>1.25	0.625	1.25	1.25
T. vulgaris *	GIZ	13.33 ± 0.57	14 ± 1	14.66 ± 0.57	22.33 ± 0.57	12.66 ± 0.57	13 ± 1
	MIC	0.078	0.156	0.156	0.156	0.156	0.156
	MBC	0.625	1.25	0.312	0.312	1.25	0.625
R. officinalis *	GIZ	-	7 ± 0	9.33 ± 0.57	12.33 ± 0.57	14 ± 0	12 ± 1
	MIC	0.31	0.195	0.625	1.25	0.156	0.156
	MBC	5	>5	>2.5	2.5	2.5	2.5
C. cyminum **	GIZ	11 ± 0	20.33 ± 0.58	15 ± 0	13.33 ± 0.58	12 ± 0	14.67 ± 0.58
	MIC	0.312	0.156	0.156	0.078	0.156	0.312
	MBC	0.625	0.625	1.25	0.625	1.25	1.25
M. pulegium *	GIZ	10 ± 1	13.33 ± 0.57	9 ± 0	11 ± 0	7 ± 0	8 ± 0
	MIC	0.097	0.78	0.39	0.195	0.39	0.195
	MBC	3.125	6.25	3.125	>1.56	3.125	3.125
M. longifolia *	GIZ	9.33 ± 1.15	7.66 ± 0.57	8.66 ± 1.15	12 ± 0	11 ± 0	11 ± 0
	MIC	0.78	0.78	0.195	0.39	0.39	0.39
	MBC	>3.125	6.25	3.125	1.56	6.25	6.25
M. spicata ***	GIZ	18.67 ± 0.58	17.67 ± 0.58	14.67 ± 0.58	15.67 ± 0.58	13.33 ± 0.58	13.33 ± 0.58
	MIC	0.023	0.047	0.023	0.023	0.023	0.023
	MBC	>24	>12	>12	>12	>6	>12

-: No inhibition; *: Snoussi *et al.*, (2008); **: Hajlaoui *et al.*, (2010); ***: This study. 1: *V*. *alginolyticus* ATCC 33787; 2: *V*. *alginolyticus* ATCC 17749; 3: *V*. *parahaemolyticus* ATCC 17802; 4: *V*. *parahaemolyticus* ATCC 43996; 5: *V*. *vulnificus* ATCC 27562; 6: *V*. *fluvialis* ATCC 33809.

Recently, Dhifi *et al.* [40] reported that the *M. spicata* essential oil (chemotype menthone/pulegone) was active against Gram+ (*S. aureus* and *S. epidermidis*), Gram- (*Salmonella* sp. and *E. coli*), and *Candida* species, with diameter of growth inhibition zones of about 20 mm against a *Salmonella* sp. strain, 18 mm against *Escherichia coli*, and 26 mm against *Candida albicans*.

Yano *et al.* [50] studied the anti-*V. parahaemolyticus* activity of 18 spices and herbs from Japan and reported that the pathogenic serotype strains O3:K6 and O4:K8 was sensitive to all spices and herbs tested. Consequently, the studied extracts can be used for protecting seafood from the risk of contamination by *V. parahaemolyticus* strains. Indeed, Shelef *et al.* [51] reported that the pathogenic strain O4:K8 and O4:K11 were more sensitive to rosemary than a non-pathogenic one. The essential oils of *Thymus daenensis* Čelak, *Satureja bachtiarica Bunge*, *Satureja khuzistanica* Jamzad, *Zataria multiflora* Boiss., *Achillea kellalensis* Boiss. & Hausskn., and *Cuminun cyminum* L., utilized as traditional medicines by the indigenous people of Chaharmahal va Bakhtiari in Iran, showed antibacterial activities against *V. parahaemolyticus* and *V. harveyi* [52]. The highest level of antibacterial activity against *V. parahaemolyticus* was demonstrated by the essential oil of *T. daenensis* (minimal inhibitory concentration (MIC = 7 µg/mL), and the highest level of antibacterial activity against *V. harveyi* was demonstrated by the essential oil of *S. bachtiarica* (MIC = 15 µg/mL).

It has been reported that when an essential oil is combined with antimicrobial agents, there is a synergistic effect against multi-drug resistant *S*. *aureus*, and in many cases, a substantial antimicrobial MIC reduction can be observed [53]. The *M. spicata* essential oil from Serbia (carvone/menthone chemotype) tested in the disc-diffusion method showed better activity against Gram+ bacteria than Gram− with a bacteriostatic activity in concentration of 1 µg/mL with diameter of inhibition zone ranging from 10 mm (*Pseudomonas aeruginosa*) to 25 mm (*Micrococcus flavus*) and MIC and MBC values ranging from 1–2.5 µg/mL and 1.5–2.5 µg/mL, respectively. Hydrocarbon monoterpenes showed the lowest bacterial activity, while oxygenated compounds resulted in a higher potential, especially phenol type compounds such as thymol and carvacrol [54,55]. On the other hand, the oxygenated monoterpenes exhibit strong antimicrobial activity pronounced on whole cell, while hydrocarbon derivates possess lower antimicrobial properties, as their low water solubility limits their diffusion through the medium [56].

It has been previously demonstrated that, l-Carvone or (*4R*)-(−)-carvone is the main component of spearmint (*Mentha spicata* Linnaeus 1753) seeds, while, d-Carvone or (*4S*)-(+)-carvone is the key component in caraway (*Carum carvi* Linnaeus 1753) and dill (*Anethum graveolens* Linnaeus 1753). This monoterpene is known for its antioxidant activity [57], antimicrobial activity [58,59], antifungal activity [60], and effectiveness as an insect repellent [61]. Carvone is used as a fragrance and flavor agent, to inhibit sprouting in stored potatoes or flower bulbs, for building blocks, and as a biochemical environmental indicator [62,63].

Uribe *et al.* [64] reported that monoterpenoids such as (*R-*) and (*S-*) carvones exert an antimicrobial effect by interacting with the microbial membrane due to their inherent lipophilicity, while, the combination of *R*- and *S*-carvone suggested that carvones have a high affinity for the bacterial cell membrane and may influence structural or functional properties of the membrane [65]. In fact, using the Transmission Electron Microscope, Mun and colleagues [66] revealed cytoplasmic disruption and separation of the cytoplasmic contents of methicillin-resistant *S. aureus* strains following exposure to *R*-carvone. Additionally, (*4R*)-(−)-carvone was also active against *Campylobacter jejuni*, *Enterococcus faecium*, *Escherichia coli*, and *Aspergillus niger* [67,68], while (*4S*)-(+)-carvone was effective against *E. coli* O157:H7, *Salmonella typhimurium Photobacterium leiognathi*, and *Listeria monocytogenes* [58,68]. In a previous work carried out by Aggarwal *et al.* [69], the results showed that the main components found in *M. spicata* oil were (*4S*)-(−)-limonene, (*4R*)-(−)-carvone), (*R*)-(+)-limonene, and (*4S*)-(+)-carvone) for *A. sowa* Roxb essential oil. They also found that both optical isomers of carvone were active against a wide spectrum of human pathogenic fungi and bacteria. Additionally, the antimicrobial properties of these monoterpenes were similar to those in spearmint and Indian dill essential oils.

### 2.4. Biofilm Inhibition and Eradication

**Figure 1:**
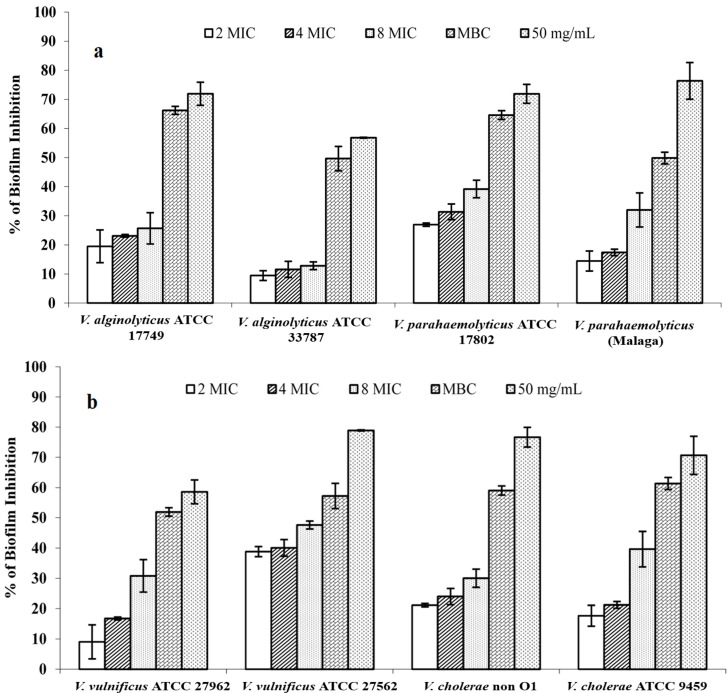
Effects of different concentrations of *M. spicata* essential oil on biofilm formation (expressed as percentage of inhibition evaluated by the XTT values) of *Vibrio* spp. strains.Errors bars represent standard deviations. Values are the average of at least three independent determinations.

**Figure 2:**
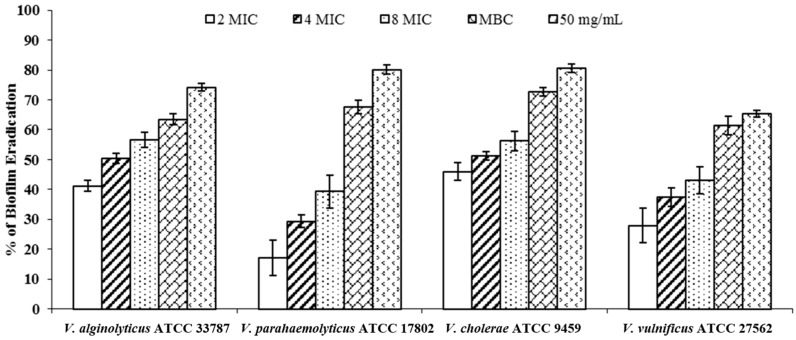
Effects of different concentrations of *M. spicata* essential oil on preformed biofilm (expressed as percentage of eradication evaluated by the XTT values) of *Vibrio* spp. strains. Errors bars represent standard deviation. Values are the average of at least three independent determinations.

The discovery of anti-infective agents, active not only against planktonic micro-organisms but also against microbial biofilms, represents an important goal. In fact, prevention of the biofilm formation effect of plant derivatives has been reported in the case of *Listeria monocytogenes*, *Pseudomonas aeruginosa*, *Streptococcus mutans*, *Staphylococcus aureus*, *Candida albicans*, and oral pathogens; however, few studies reported the same effect on pathogenic *Vibrio* strains [70]. In this study we tested the ability of different concentrations of *M. spicata* essential oil to inhibit or eradicate biofilm on a polystyrene surface formed by four pathogenic *Vibrio* spp. species, *V. vulnificus*; *V. cholerae*; *V. parahaemolyticus*, and *V. alginolyticus*. Our results showed that spearmint essential oil inhibited the tested *Vibrio* spp. Strains’ biofilm production by 11.5% and 11.6% for *V. alginolyticus* ATCC 33787, and by 28% and 40% for *V. vulnificus* ATCC 27562 at 0.046 and 0.092 mg/mL, respectively (Figure 1). As regards the *Vibrio* preformed biofilm disruption (Figure 2), the spearmint essential oil eradicated more than 50% of preformed *V. cholerae* ATCC 9459 and *V. alginolyticus* ATCC 3378 biofilms at 0.092 mg/mL. The fact that any activity was reliable only at concentrations higher than the MIC values leads us to believe that such activities are due to the bacteriostatic activity of the essential oil rather than to the presence of compounds affecting the biofilm formation.

Similar results were obtained with different essential oils of *Mentha* species [71,72,73]. Quave *et al.* [74] studied the effect of *M. spicata* (ethanolic extracts) on growth and biofilm formation in a methicillin-resistant *Staphylococcus aureus* (ATCC 33593). In 2009, Rasooli and coworkers [75] studied the chemical composition and the effect of Iranian *M. spicata* essential oil on dental biofilm and reported that the oil (chemotype limonene/piperitone) was active against caryogenic bacteria with a diameter of zone inhibition of about 60 mm against *Streptococcus mutans* and 46 mm against *S. pyogenes*, and that it retards biofilm formation. Moreover, Mousavi *et al.* [76] found that the MIC of *M. spicata* essential oils against *P. aeruginosa* was 16 μg/mL. They also showed that all essential oils tested at 1/2 and 1/4 MIC significantly reduced all *P. aeruginosa* virulence factors tested. At 1/8 MIC, *M. spicata* oil had effect just on adhesion, but *Cuminum cyminum* oil had effect on alginate production, biofilm formation, swimming, and twitching.

Recently, Karthikeyan *et al.* [77] tested the antibiofilm and anti-quorum sensing activity of the leaf extract of *Dendrophthoe falcate* (L.f.) Ettingsh (Loranthaceae) against different bacterial pathogens. They reported that among the 17 bacterial pathogens screened, the methanolic fraction of the leaf extract clearly demonstrated antibiofilm activity against *Proteus mirabilis*, *P. vulgaris*, *Vibrio vulnificus*, *V. parahaemolyticus*, *V. harveyi*, *V. alginolyticus*, *V. cholerae*, *Aeromonas hydrophila*, *Sighella sonnei*, and *Chromobacterium violaceum* ATCC 12472. At the biofilm inhibitory concentrations, biofilm formation was reduced by up to 70%–90%.

## 3. Experimental Section

### 3.1. Plant Material and Extraction of Essential Oil

*Mentha spicata* plants were freshly purchased in December 2010 from the Soliman Tunisian locality (Nabeul) and identified according to the flora of Tunisia [1] by Professor Abderrezak Smaoui from the Center of Biotechnology (Technopark of Borj Cédria, Tunisie). A voucher specimen (SM-1) was deposited in the laboratory of Wastewaters Treatment of the Biotechnology Centre. The aerial parts were dried at room temperature. One hundred grams of material sample were subjected to hydrodistillation for 3 h with 500 mL of distilled water using a Clevenger-type apparatus according to the *European Pharmacopoeia* [78]. This step was repeated many times to obtain a considerable volume of essential oil (10 mL), then dried over anhydrous sodium sulfate and stored in sealed glass vials in a refrigerator at 4 °C prior to analysis. The density of the essential oil obtained was 0.96 (10 µL of essential oil weighted 9.6 mg).

### 3.2. GC-EIMS Analysis

GC-EIMS analyses were performed with a Varian CP-3800 gas-chromatograph equipped with a HP-5 capillary column (30 m × 0.25 mm; coating thickness 0.25 μm) and a Varian Saturn 2000 ion trap mass detector. Analytical conditions: injector and transfer line temperatures at 220 and 240 °C, respectively; oven temperature was programmed from 60 to 240 °C at 3 °C/min; carrier gas helium at 1 mL/min; injection of 0.2 µL (10% hexane solution); split ratio 1:30. Identification of the constituents was based on comparison of the retention times with those of authentic standards, comparing their Linear Retention Indices relative to the series of *n*-hydrocarbons, and by computer matching against commercial libraries (NIST 98 and ADAMS 95) and a home-made library of mass spectra built up from pure substances and components of known essential oils and MS literature data [79,80,81,82,83,84]. Linear retention indices have been calculated using the *n*-alkanes series (C8–C23) using the Van den Dool and Kratz formula [85]. Moreover, the molecular weights of all the identified substances were confirmed by chromatography chemical ionization mass spectrometry (GC-CIMS), using MeOH as a CI ionizing gas.

### 3.3. Antioxidant Properties

#### 3.3.1. DPPH Radical-Scavenging Activity.

The DPPH radical scavenging activity was evaluated according to the method described by Hajlaoui *et al.* [16] with some modifications. Briefly, 0.25 mL of a 0.2 mM DPPH• methanolic solution was mixed with 1 mL of essential oil at different concentrations (0.01, 0.02, 0.1, and 0.2 mg/mL) or with 1 mL of control sample. The mixture was left for 30 min at room temperature in the dark. The absorbance was measured at 515 nm and the scavenging activity (SA%) against DPPH radicals was calculated using the following equation:

SA% = [(A_c_ − A_s_)/A_c_] × 100 (1)

where A_c_ is the absorbance of the control at 30 min and A_s_ is the absorbance of the sample at 30 min; a BHT solution at different concentrations (1–50 µg/mL) was also tested. IC_50_ values represented the essential oil and BHT concentrations (µg/mL) scavenging 50% of DPPH radicals. All samples were analyzed in triplicate.

#### 3.3.2. Metal Chelating Activity.

Ferrous ion chelating activity was estimated as described by Ksouri *et al.* [86]. Briefly, different concentrations of essential oil (1, 5, and 15 mg/mL) were added to 0.05 mL of 2 mM FeCl_2_·4H_2_O solution and left for incubation at room temperature for 5 min. Afterwards, the reaction was initiated by adding 0.1 mL of 5 mM ferrozine and the mixture was adjusted to 3 mL with deionized water, shaken vigorously, and left standing at room temperature for 10 min. The absorbance of the solution was then measured at 562 nm. EDTA (5–100 µg/mL) was used as a positive control. The percentage of inhibition of ferrozine-Fe^2^^+^ complex formation was calculated using the following equation:

Metal chelating activity MCA (%) = [(A_c_ − A_s_)/A_c_] × 100 (2)

where A_c_ is the absorbance of the control and A_s_ is the absorbance of the sample (essential oil or EDTA standard solutions). Results were expressed as IC_50_. The IC_50_ values are the concentrations required to chelate 50% of ferrous ions present in the system. Analyses were run in triplicates.

#### 3.3.3. Determination of Reducing Power

The ability of the spearmint essential oil to reduce Fe^3+^ was assayed by the method of Hajlaoui *et al.* [16]. Briefly, 1 mL of spearmint essential oil was mixed with 2.5 mL of phosphate buffer (0.2 M, pH 6.6) and 2.5 mL of K_3_Fe(CN)_6_ solution (1 g/100 mL). The mixture was incubated at 50 °C for 25 min, then 2.5 mL of a trichloroacetic acid solution (10 g/100 mL) was added and the mixture was centrifuged for 10 min at 650× *g*. Finally, 2.5 mL of the upper layer was mixed with 2.5 mL of distilled water and 0.5 mL of FeCl_3_ aqueous solution (0.1 g/100 mL). The absorbance of the mixture was measured at 700 nm. A higher absorbance of the reaction mixture indicated a higher reducing power. A standard curve was generated using ascorbic acid (10–100 µg/mL), plotting the mean absorbance values against ascorbic acid concentrations, and a linear regression analysis was carried out. The EC_50_ value (µg/mL) is the effective concentration at which the absorbance was 0.5 for the reducing power. Ascorbic acid was used as a positive control.

#### 3.3.4. Anti-Peroxyl Radical Activity.

The anti-peroxyl radical activity was evaluated measuring the peroxides generated during the oxidation of linoleic acid at high temperature, according to the method of Hajlaoui *et al.* [16] with some modifications. Briefly, 0.2 mg of β-carotene was dissolved in 2 mL of chloroform, and added to 20 mg of linoleic acid and 200 mg of Tween 40. After removing CHCl_3_ under a vacuum, oxygenated water (100 mL) was added and the flask was vigorously shaken until all material dissolved. The emulsion obtained was freshly prepared before each experiment. An aliquot of 150 μL of emulsion was distributed in each of the wells of 96-well microtiter plates and 10 mg of essential oil or BHA standard solution (0.1–100 µg/mL) was added. An equal amount of emulsion was used for the blank sample. The microtiter plate was incubated at 45 °C and the absorbance was measured at 490 nm using a visible/UV microplate kinetics reader (EL x 808, Bio-Tek instruments, Winooski, VT, USA). Readings of all samples were performed immediately (*t* = 0 min) and after 120 min of incubation. The antioxidant activity (AA) of the essential oil was evaluated in term of β-carotene blanching using the following equation:

AA% = [(A_0_ − A_t_)/A_0_] × 100 (3)

where A_0_ is the absorbance of the control at 0 min and A_t_ is the absorbance of the sample (essential oil or BHA) at 120 min. The results are expressed as IC_50_ values (μg/mL). The IC_50_ values are the concentrations required to inactivate 50% of the preformed peroxyl radicals. All samples were analyzed in triplicate.

### 3.4. Antimicrobial Activity

#### 3.4.1. Microorganisms

The antibacterial effect of the essential oil was evaluated against 30 strains belonging to *Vibrio* genus (16 different species) and against *Aeromonas hydrophila* ATCC 7966^T^. These microorganisms were previously isolated from diseased *Sparus aurata*, *Dicentrarchus labrax*, and *Mytilus edulis* in Tunisia [15] and the type strains were kindly provided by Professor Stefania Zanetti from the Department of Biomedical Sciences (University of Sassari, Sassari, Italy), Professor Jesús López Romalde from the Department of Microbiology and Parasitology (CIBUS-Facultad de Biologia, Universidad de Santiago, Santiago de Compostela, Spain), Professor Donatela Ottaviani from the Italian Reference Center for Microbiological and Chemical Control on Shellfish-State Veterinary Institute for Umbria and the Marches (IZSUM, Ancona, Italy), Professor Miguel Angel Morinigo from the Department of Microbiology (Facultad de ciencia de Malaga, Campus de Teatinos, Spain) and Professor Bruno Gomez Gil (Mazatlán Unit for Aquaculture, Sinaloa, Mexico).

#### 3.4.2. Disk-Diffusion Assay

The antimicrobial activity test was done according to the protocol described by Snoussi *et al.* [15] for *Vibrio* spp. strains. For the experiments, a loopful of the microorganisms working stocks were enriched on a tube containing 9 mL of Mueller-Hinton broth supplemented with 1% NaCl then incubated at 37 °C for 18–24 h. The overnight cultures were used for the antimicrobial activity of the essential oils used in this study and the optical density was adjusted at 0.5 (OD_520nm_). The inocula were streaked onto Mueller Hinton 1% NaCl agar plates; then the sterile filter discs (diameter 6 mm, Biolife, Milano, Italy) were impregnated with 10.4 µL of essential oil (10.4 µL of essential oil weighed 10 mg).

Five antibiotics were used in this study as positive controls for *Vibrio* spp. strains. The antibiotic susceptibility was determined using the Kirby-Bauer method and Mueller-Hinton agar plates supplemented with 1% NaCl, as described by Ottaviani *et al.* [87]. The dishes were incubated at 37 °C for 18–24 h for microbial strains. The diameter of the zones of inhibition was measured with 1 mm flat rule. Each experiment was carried out in triplicate and the mean diameter of the inhibition zone was recorded.

#### 3.4.3. Micro-Well Determination of MIC and MBC

The minimal inhibition concentration (MIC) and the minimal bactericidal concentration (MBC) values were determined for all *Vibrio* spp. strains used in this study as described by Gulluce *et al.* [88] and Snoussi *et al.* [15]. The inocula of the bacterial strains were prepared from overnight cultures and suspensions were adjusted to 0.1 standard turbidity (OD_600nm_). The essential oil dissolved in 10% dimethylsulfoxide (DMSO) was diluted to the highest concentration (48 mg/mL) to be tested and then serial twofold dilutions were made in the concentration range 0.023–24 mg/mL in the 96-well plates. In fact, the 96-well plates were prepared by dispensing into each well 95 µL of Mueller-Hinton 1% NaCl broth. Then, 100 µL of the highest concentration (48 mg/mL) were added to the first well and consecutively two-fold dilutions were prepared in the next 10 wells. Finally, 5µL of the inoculum of each microorganism were added to the wells. The last well, containing 195 µL of Mueller-Hinton 1% NaCl broth without essential oil and 5 µL of the bacterial inoculum of each strain, was used as the negative control. The final volume in each well was 200 µL. The plates were incubated at 37 °C for 18–24 h.

The MIC was defined as the lowest concentration of the sample that did not permit any visible growth of the tested microorganism after incubation at 37 °C as compared to the control well (grown without essential oil), whereas the MBC value was determined by subculture of 10 µL from the wells medium with no visible growth onto Mueller–Hinton agar plates. After 24 h of incubation at 37 °C, the growth of the microorganism was observed. When no growth was observed, the sample denoted a bactericidal action.

### 3.5. Assessment of Vibrio spp. Biofilm Metabolic Activity Using XTT Reduction Assay

The ability of *Vibrio* spp. strains to form a biofilm on polystyrene surface was quantified using the XTT [2,3-bis(2-methyloxy-4-nitro-5-sulfophenyl)-2*H*-tetrazolium-5-carboxanilide] reduction assay, according to methods described previously by Chaieb *et al.* [89]. This technique measures the metabolic activity of cells in biofilm by measuring the reduction of a tetrazolium salt by metabolically active cells to a colored water-soluble formazan derivative easily quantified calorimetrically. Eight *Vibrio* spp. strains (*V. alginolyticus* ATCC 17749, *V. alginolyticus* ATCC 33787, *V. parahaemolyticus* ATCC 17802, *V. parahaemolyticus* (Malaga), *V. vulnificus* ATCC 27962, *V. vulnificus* ATCC 27562, *V. cholerae* non O1 (IPT), and *V. cholerae* ATCC 9459) were grown overnight in Brain Infusion Broth (BHI-0.25 glucose at 37 °C). The culture was diluted 1:20 in fresh BHI-1% NaCl plus (0.25%) glucose at 37 °C. Two hundred μL of this suspension were used to inoculate sterile 96-well-polystyrene microtiter plates (Nunc, Roskilde, Denmark). XTT (Sigma-Aldrich, Buchs, Switzerland) solution (1 mg/mL) was prepared in PBS, filter sterilized, and stored at −80 °C. Menadione (Sigma-Aldrich) solution (1 mM) was prepared in acetone and sterilized immediately before each assay. Following incubation, the biofilms were first washed five times with PBS, and then 100 μL PBS and 12 μL XTT–menadione solution (12.5:1 *v*/*v*) were added to each of the prewashed wells and the control wells. The plate was then incubated for 3 h in the dark at 37 °C. Following incubation, 100 μL of the solution was transferred to fresh wells, and the color change in the solution was measured with a Multiskan reader at 490 nm (BioRad, Marnes-la-Coquette, France). The absorbance values for the controls were then subtracted from the values of the tested wells to eliminate spurious results due to background interference. Each assay was repeated three times.

### 3.6. Effect of Essential Oils on Vibrio spp. Biofilm Formation

*Mentha spicata* essential oil was tested for its potential to inhibit and/or eradicate biofilm formation of four type strains including: *V. alginolyticus* ATCC 33787, *V. parahaemolyticus* ATCC 17802, *V. vulnificus* ATCC 27962, and *V. cholerae* ATCC 9459 as previously described by Nostro *et al.* [90] with some modifications. The essential oils was added to the growth medium (BHI-1% NaCl plus 0.25% glucose) at the time of inoculation and the cells were allowed to form biofilms. Prevention of biofilm formation was examined by microdilution, similar to the MIC assay for planktonic cells. A two-fold serial dilution was prepared in 96-well polystyrene tissue culture plates containing BHI-1% NaCl plus 0.25% glucose with final concentrations of essential oil corresponding to 2 MIC, 4 MIC, 8 MIC, MBC, and >MBC (50 mg/mL). The medium without essential oil was used as a control. Each assay was repeated three times. The XTT assay was also used to quantify the biofilm formed. In order to access the ability of the essential oil to prevent biofilm formation, the percentage of biofilm inhibition was calculated using the equation:

[(OD(growth control) − OD(sample))/OD(growth control)] × 100
(4)

### 3.7. Effect on Established Biofilms

The effect on established biofilms of the obtained essential oil was verified as described by Nostro *et al.* [90] with some modifications. All *Vibrio* spp. strains were grown overnight in Brain Infusion Broth (BHI-0.25 glucose) at 37 °C. The culture was diluted 1:20 in fresh BHI-1% NaCl plus 0.25% glucose at 37 °C. Two hundred microliters of this suspension were used to inoculate sterile 96-well-polystyrene microtiter plates (Nunc, Roskilde, Denmark). Following incubation, the biofilms were first washed five times with PBS, and then 200 μL of the essential oil prepared in BHI-1% NaCl plus 0.25% glucose were added to each prewashed and control wells. The microplates were incubated for 24 h at 37 °C. Following incubation, the wells were first washed five times with PBS and then 200 µL of PBS and 12 μL of XTT–menadione solution (12.5:1 *v*/*v*) were added to each well. The plate was then incubated for 3 h in the dark at 37 °C and the color change in the solution was measured with a Multiskan reader at 490 nm (BioRad, Tokyo, Japan). The absorbance values for the controls were then subtracted from the values of the tested wells to eliminate spurious results due to background interference. Each assay was repeated three times. In order to access the ability of the essential oil to treat preformed mature biofilms, the percentage of biofilm eradication was calculated using the equation:

[(OD(growth control) − OD(sample))/OD(growth control)] × 100
(5)

### 3.8. Statistical Analysis

All analyses were performed in triplicate and the results are expressed as mean values ± standard deviations (SD). The data were subjected to statistical analysis using the statistical program package STATISTICA (Statsoft, 1998, Tulsa, OK, USA). The differences in mean were calculated using Duncan’s multiple range tests for means with 95% confidence limit (*P* = 0.005).

## 4. Conclusions

The reported results obtained for Tunisian *M. spicata* essential oil (carvone/limonene chemotype) showed high antibacterial activity against *Vibrio* spp. as well as antioxidant properties. The low MIC and MBC values indicated that its use could consistently contribute to preserve seafood from the *Vibrio* proliferation that makes particularly hazardous the spreading worldwide habit of eating such foods raw or not well done.

Furthermore, the high antioxidant and anti-free radical activities registered could be important to preserve marine products known to contain a number of components prone to degradation. In particular, polyunsaturated fatty acids that quickly undergo peroxidation, generating as a primary product the peroxyl radicals, could be efficiently scavenged by the *M. spicata* essential oil. Therefore, such properties could be useful in prolonging seafood’s shelf life because they are protective against both microbiological and chemical deterioration, thus preventing loss of flavor and, most importantly, toxic agent formation.

Furthermore, it should be noted that, when ingested, *M. spicata* essential oil—due to its antioxidant, antiradical, and chelating properties—could contribute, with different mechanisms, to maintaining the balance of the organism’s redox status. Hence, the use of *M. spicata* and its essential oil as a seafood seasoning could be useful in improving the seafood product’s taste, safety, and effect on human health.
